# Supplementary material for: Diabetes Related Distress in Children with Type 1 Diabetes before and during the COVID-19 Lockdown in Spring 2020
Source: Int J Environ Res Public Health. 2021 Aug 12;18(16):8527. doi: 10.3390/ijerph18168527 (PMC8394974; doi:10.3390/ijerph18168527)
Supplement: Supplementary file 1 [file ijerph-18-08527-s001.zip › ijerph-1316028-supplementary.pdf]

Supplementary materials, Questionnaires
(manuscript title: *Diabetes related distress in Polish children with type 1 diabetes before and during the COVID-19 lockdown in spring 2020*)

1. PAID-Ch, P-PAID-Ch, PAID-T, P-PAID-T questionnaires - INFO

We obtained official permissions to use PAID questionnaires in our study from PAID coauthors.
PAID-Ch (Problem Areas in Diabetes – Child version) 11-item questionnaire, P-PAID-Ch (Problem Areas in Diabetes - Parents of Children) 16-item questionnaire, PAID-T (Problem Areas in Diabetes – Teen version) 14-item questionnaire and P-PAID-T (Problem Areas in Diabetes - Parents of Teens) 15-item questionnaire were used.
Problem areas covered by PAID questionnaires include: emotional burden, diabetes therapy and regimen-specific burden, family and friends-related distress.
All four versions of PAID questionnaires were translated from English into Polish by one translator, and the reverse Polish-English translation (prepared by the second translator) was independently verified by the third translator to validate and confirm compatibility of content of both versions.

2. Semi-open questionnaire concerning the impact of the COVID-19 pandemic on diabetes care

In the questionnaire version below (translated from Polish into English) we added, which questions were related to *Difficulties*, which to *Worries/Concerns*. It was not stated in the original Polish version used in the study. This Questionnaire was filled-in by researchers during their phone-calls with respondents (parents of children with T1DM). Answers 1 and 2 were regarded as negating/negative, 3 as neutral/indifferent, 4 and 5 as confirmative.

****

Impact of COVID-19 pandemic in Poland on the quality of life and distress of children and adolescents with type 1 diabetes and their families

Patient's name and surname: .... Date of completion: ....

Method of completion: A. a phone call with mother/father ………. (more than 1 person is possible to check) B. sent by e-mail *(was not used)*

Questions in this survey concern what your and your child's life with diabetes has been like for the last 4-6 weeks (since 12th of March). Your answers will help us understand how you have been managing it in recent times – what works well and in which areas you require support. There are no good or bad answers. When answering the questions, please consider what your last weeks have been like. For each question, please select the most suitable answer which describes your and your child's situation the best. In the following questions, please choose on a scale from 1 to 5 to what extent you agree with the given statement (1 – I strongly disagree, 5 – I strongly agree).

Difficulties

1. Regarding the current COVID-19 pandemic situation, taking care of the child with diabetes has become more difficult

1<br>I strongly disagree	2<br>I somewhat disagree	3<br>I cannot decide	4<br>I somewhat agree	5<br>I strongly agree

2. Have you had difficulties with access to medications or medical equipment required to treat your child's diabetes?

1<br>I strongly disagree	2<br>I somewhat disagree	3<br>I cannot decide	4<br>I somewhat agree	5<br>I strongly agree

3. Have you had difficulties contacting the Diabetology Clinic?.

1<br>I strongly disagree	2<br>I somewhat disagree	3<br>I cannot decide	4<br>I somewhat agree	5<br>I strongly agree

4. Have you had difficulties contacting your diabetologist?

1<br>I strongly disagree	2<br>I somewhat disagree	3<br>I cannot decide	4<br>I somewhat agree	5<br>I strongly agree

5. Would you find teleconsultation with your diabetologist helpful in taking care of your child?

1<br>I strongly disagree	2<br>I somewhat disagree	3<br>I cannot decide	4<br>I somewhat agree	5<br>I strongly agree

6. Have you had difficulties contacting a primary care doctor?

1<br>I strongly disagree	2<br>I somewhat disagree	3<br>I cannot decide	4<br>I somewhat agree	5<br>I strongly agree

7. Others – if you face any other significant difficulties, that you would like to draw our attention to, please list them below:

…….

Worries/Concerns

8. Regarding the current COVID-19 pandemic situation, has taking care of your child with diabetes become more stressful than before?

1<br>I strongly disagree	2<br>I somewhat disagree	3<br>I cannot decide	4<br>I somewhat agree	5<br>I strongly agree

9. Are you concerned about a possible shortage of medications and medical equipment in pharmacies and wholesalers needed to treat diabetes?

1	2	3	4	5

I strongly disagree	I somewhat disagree	I cannot decide	I somewhat agree	I strongly agree

10. Are you concerned that the course of COVID-19 could be more severe in a child with diabetes?

1<br>I strongly disagree	2<br>I somewhat disagree	3<br>I cannot decide	4<br>I somewhat agree	5<br>I strongly agree

11. Are you concerned that the medical staff at infectious disease hospitals could be inadequately trained in terms of taking care of a child with diabetes?

1<br>I strongly disagree	2<br>I somewhat disagree	3<br>I cannot decide	4<br>I somewhat agree	5<br>I strongly agree

12. Are you concerned about the difficulties in ensuring your child a proper care in case you get sick?

1<br>I strongly disagree	2<br>I somewhat disagree	3<br>I cannot decide	4<br>I somewhat agree	5<br>I strongly agree

13. Are you concerned about a possible stay in the hospital's diabetology department during COVID-19 pandemic in case of acute exacerbation of your child's diabetes?

1<br>I strongly disagree	2<br>I somewhat disagree	3<br>I cannot decide	4<br>I somewhat agree	5<br>I strongly agree

14. Others – if there is anything else that concerns you about the current situation, please list it below:

……

****
